# Identification of Genes Predicting Poor Response of Trastuzumab in Human Epidermal Growth Factor Receptor 2 Positive Breast Cancer

**DOI:** 10.1155/2022/9529114

**Published:** 2022-07-27

**Authors:** Xinrui Dong, Huijuan Dai, Aijun Sun, Zhenfeng Yu, Yueyao Du

**Affiliations:** ^1^Department of Breast Surgery, Renji Hospital, School of Medicine, Shanghai Jiaotong University, 1630 Dongfang Road, 200127 Shanghai, China; ^2^Department of Thyroid and Breast Oncological Surgery, Xuzhou Medical College Affiliated Huaian Hospital, 62 Huaihai South Road, Huaian, Jiangsu 223001, China; ^3^Department of General Surgery, Shanghai Fengxian Central Hospital, 6600 NanFeng Road, 201499 Shanghai, China

## Abstract

**Objective:**

To identify trastuzumab-resistant genes predicting drug response and poor prognosis in human epidermal growth factor receptor 2 positive (HER2+) breast cancer.

**Methods:**

Gene expression profiles from the GEO (Gene Expression Omnibus) database were obtained and analyzed. Differentially expressed genes (DEGs) between the pathological complete response (pCR) group and non-pCR group in a trastuzumab neoadjuvant therapy cohort and DEGs between Herceptin-resistant and wild-type cell lines were detected and evaluated. Gene Ontology (GO) and Kyoto Encyclopedia of Genes and Genomes (KEGG) pathways analyses were performed to select the functional hub genes. The hub genes' prognostic power was validated by another trastuzumab adjuvant treatment cohort.

**Results:**

Fifty upregulated overlapping DEGs were identified by analyzing two trastuzumab resistance-related GEO databases. Functional analysis picked out ten hub genes enriched in mitochondrial function and metabolism pathways: *ASCL1*, *CPT2*, *DLD*, *ELVOL7*, *GAMT*, *NQO1*, *SLC23A1*, *SPR*, *UQCRB*, and *UQCRQ.* These hub genes could distinguish patients with trastuzumab resistance from the sensitive ones. Further survival analysis of hub genes showed that *DLD* overexpression was significantly associated with an unfavorable prognosis in HER2+ breast cancer patients.

**Conclusion:**

Ten novel trastuzumab resistance-related genes were discovered, of which *DLD* could be used for trastuzumab response prediction and prognostic prediction in HER2+ breast cancer.

## 1. Introduction

HER2 (human epidermal growth factor receptor 2) positive breast cancer accounts for 20-30% of the total primary breast cancer population [1]. HER2 positive (HER2+) patients were traditionally correlated with the poor outcome before the discovery of trastuzumab, a monoclonal antibody targeting to suppress HER2 activity [2]. Until now, trastuzumab is still the first-line therapeutic drug for treating HER2+ breast cancer patients [3]. Trastuzumab can immensely increase the clinical prognosis of primary HER2+ breast cancer and metastatic HER2+ breast cancer [4]. However, the resistance of trastuzumab is a major impediment to impairing its efficacy for patients. So, further identification of genomic alterations of trastuzumab insensitivity is crucial to provide potential biomarkers for precise treatment of trastuzumab and to dig novel therapeutic strategies.

Gene profiling and signatures are utilized to quickly detect differentially expressed genes (DEGs), which have significantly promoted the progress of tumor research during the past decades. A few studies have analyzed the predictive and prognostic value of transcript sequencing-based genes from breast cancer patients. Through analysis of genome-wide expression profiling, Sotiriou et al. found out that topoisomerase II-alpha (*TOP2A*) expression could effectively predict the response of anthracycline (epirubicin) for estrogen receptor– (ER–) negative breast cancer patients who received neoadjuvant chemotherapy (NCT) [5]. Additionally, a 4-microRNAs-based signature was found to be of significant value in predicting the pathological complete response (pCR) for basal-like triple negative breast cancer (TNBC) breast cancer patients [6], and a 17-gene signature was found to have an excellent predictive and prognostic role in TNBC patients who receive anthracycline-based NCT [7]. However, a few studies were conducted to identify gene signatures predicting trastuzumab sensitivity for HER2+ patients. Hence, we performed a profound analysis using integrated bioinformatic methods for massive public data. Ultimately, we identified essential genes that could predict the therapeutic sensitivity of trastuzumab in HER2+ patients, which is of great clinical significance.

With the widespread application of high-throughput techniques, GEO (Gene Expression Omnibus) provides a platform that includes millions of datasets, tissues, and cell samples to analyze public gene expression. This allowed gene analysis from discovering molecular biomarkers and classifying disease by comparing phenotypes. Our study first calculated the DEGs by drug response in a trastuzumab neoadjuvant treatment cohort in GEO databases. We also found another DEG cluster between Herceptin-resistant and wild-type cell lines. We identified an upregulated gene set with DEGs based on the overlapping analysis. Several functional evaluations revealed that 10 DEGs were related to mitochondria, identified as hub genes. The survival analysis of the hub genes in another trastuzumab adjuvant cohort demonstrated that only *DLD* overexpression was significantly associated with poor outcomes. Further survival and immune studies explored that *DLD* may be discovered as a biomarker for trastuzumab response prediction and HER2+ breast cancer prognosis assessment.

## 2. Methods

### 2.1. Data Collection

The NCBI- (National Center for Biotechnology Information-) GEO datasets GSE62327, GSE15043, and GSE58984 were searched using “trastuzumab” and “breast cancer” as keywords. GSE62327 contains the RNA-seq data and clinical information, especially drug responses to different neoadjuvant therapy. We chose 6 patients with pCR and 18 patients with non-pCR in patients who used trastuzumab as neoadjuvant therapy. GSE15043 comprises the RNA-seq data of 8 Herceptin-resistant breast cancer cell lines and 2 wild-type breast cancer cell lines. GSE58984 has the RNA-seq data and clinical information from a cohort of 94 patients who used trastuzumab as adjuvant therapy. All microarray data were obtained from the GEO database: https://www.ncbi.nlm.nih.gov/geo/. The raw data were downloaded as MINiML files.

The RNA-sequencing data of all upregulated genes and corresponding clinical information were downloaded from the cancer genome atlas (TCGA) dataset (https://portal.gdc.com) in HER2+ breast cancer despite the status of hormone receptors. Finally, 185 samples were chosen. Counting data is converted to transcripts per million (TPM) and normalized log2 (TPM+1) while keeping clinical information intact.

### 2.2. Identification of DEGs (Differentially Expressed Genes)

The “limma” R package was used to compare the genome expression profile of GSE62327 and GSE15043. In GSE62327, DEGs between the pCR and non-pCR group with a false discovery rate (FDR) <0.05 were picked. In GSE15043, DEGs between wild-type and Herceptin-resistant breast cancer cell lines with an FDR <0.05 were chosen. DEGs associated with overall survival (OS) were assessed using a univariate Cox proportional hazard regression model analysis. Volcano plots and forest plots were constructed using ggplot2 packages. The overlapping DEGs were drawn in a Venn diagram using the “VennDiagram” R package.

### 2.3. Functional Enrichment Analysis

The “clusterProfiler” R package was used to conduct Gene Ontology (GO), Kyoto Encyclopedia of Genes and Genomes (KEGG) pathways analysis, and GO and KEGG pathways' network figure. The network's top nodes with the most connectivity lines were identified as hub genes.

### 2.4. Construction of PPI (Protein-Protein Interaction) Network

The PPI networks for overlapping DEGs were conducted by the STRING database (version 11.5) and Cytoscape software (version 3.9.1). The top nodes with mitochondrial function were identified as hub genes.

### 2.5. Mitochondrial Proteins and Pathways Annotation

The annotations of mitochondrial-related genes were interpreted by MitoCarta3.0 downloaded from https://pubs.broadinstitute.org/mitocarta/mitocarta30-inventory-mammalian-mitochondrial-proteins-and-pathways.

### 2.6. Validation and Screening of Hub Genes

To confirm the prognostic effect of hub genes in patients using trastuzumab as adjuvant therapy, survival analyses were carried out in GSE58984 using the hub genes' profile. The distant disease-free survival (DDFS) of each hub gene was calculated by Kaplan-Meier analysis with the log-rank test. The Kaplan-Meier curves were drawn using the “surviminer” R package.

### 2.7. Estimate the Proportion of Immune and Cancer Cells (EPIC)

Estimation of immune fractions of *DLD* was determined through HER2+ breast tissue in TCGA database using EPIC by R package “GfellerLab/EPIC.” The student *t*-test was calculated and visualized using R software.

### 2.8. Statistical Analysis

Wilcoxon rank-sum test was used to compare the gene expression between two groups. Kruskal-Wallis test was used to compare the gene expression among different groups. The OS, disease-specific survival (DDS), and progress-free interval (PFI) between other groups were calculated by Kaplan-Meier analysis with the log-rank test. Spearman's correlation analysis was used to describe the correlation between quantitative variables. All statistical analyses were performed with R software (Version 4.1.3). All *P* values were two-tailed unless otherwise specified, and *P* values less than 0.05 were considered statistically significant.

## 3. Results

### 3.1. Identify DEGs in Trastuzumab Neoadjuvant Therapy Cohort and Herceptin-Resistant Cells

**Figure 1:**
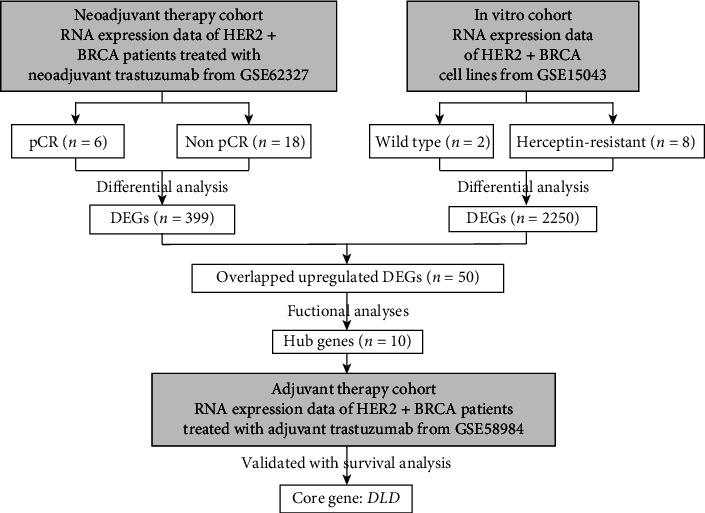
Flow chart of data collection and screening of trastuzumab-resistant gene *DLD.* GSE62327 and GSE15043 were selected to find potential trastuzumab resistance genes. Then, we carried out the functional analysis and figured out ten hub genes. GSE58984 was chosen as the prognostic validation cohort of hub genes. Finally, *DLD* was discovered. The detail of data collection was demonstrated in the methods part.

**Figure 2:**
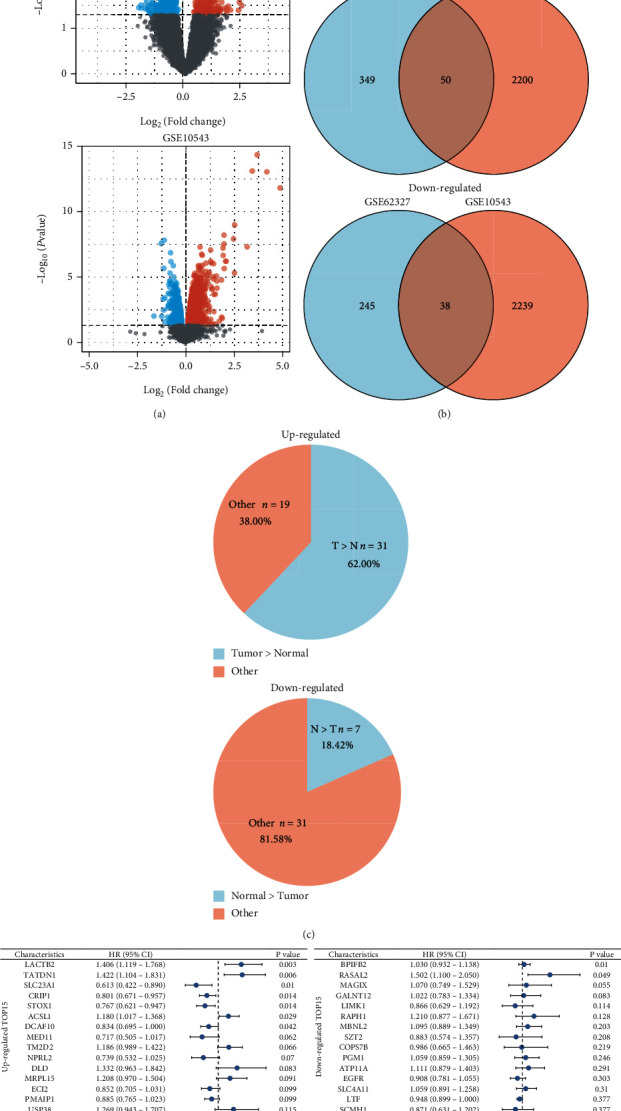
Demonstration of the DEGs in 2 GEO datasets and identification of overlapping genes. (a) Volcano plot of DEGs in GSE62327 and GSE5043. Red dots: upregulation; blue dots: downregulation; grey dots: non-differentially expressed genes. (b) Venn diagram to identify the common upregulated and downregulated DEGs in two cohorts. (c) Pie charts to compare expression in tumor tissues and adjacent nontumorous tissues in 50 upregulated DEGs and 38 downregulated DEGs separately. (d) Forest plots to demonstrate the univariate Cox regression analysis results between DEGs expression and OS. The most significant 15 DEGs were shown. DEGs: differentially expressed genes.

Twenty-four patients from GSE62327 were assigned to the “pCR” group (*n* = 6) and “non pCR” group (*n* = 18) by the response to trastuzumab monotherapy as neoadjuvant therapy at the pathological level (Figure 1). The detailed clinical information of these patients was summarized (Supplementary Table 1). In another queue, 10 cell lines from GSE15043 were divided into the “wild-type” group (*n* = 2) and the “Herceptin-resistant” group (*n* = 8) (Figure 1). We compared the whole-genome expression profile between groups in each cohort. A total of 399 DEGs from GSE62327 and 2250 DEGs from GSE15043 were detected, respectively (Table 1). The volcano plots of the DEGs were presented in Figure 2(a) (*p* < 0.05). The Venn diagrams (Figure 2(b)) obtained 50 upregulated DEGs and 38 downregulated DEGs in common. Most upregulated overlapping DEGs (31/50, 62%) were overexpressed in tumor tissues compared to adjacent nontumorous tissues in TCGA datasets (Figure 2(c)). In turn, the majority of the downregulated overlapping DEGs (31/38, 81.56%) had a lower expression level in tumor tissues (Figure 2(c)). The univariate Cox proportional hazard regression analysis of all overlapping DEGs was performed (Figures S1(a)–S1(b)). The trastuzumab resistance-related DEGs did not provide a unified prognostic value in each group. There were more significant results in the upregulated group than downregulated ones (Figure 2(d)), so we focused our attention on upregulated genes.

### 3.2. Conduct Functional Analysis of Upregulated DEGs and Find out Hub Genes

**Figure 3:**
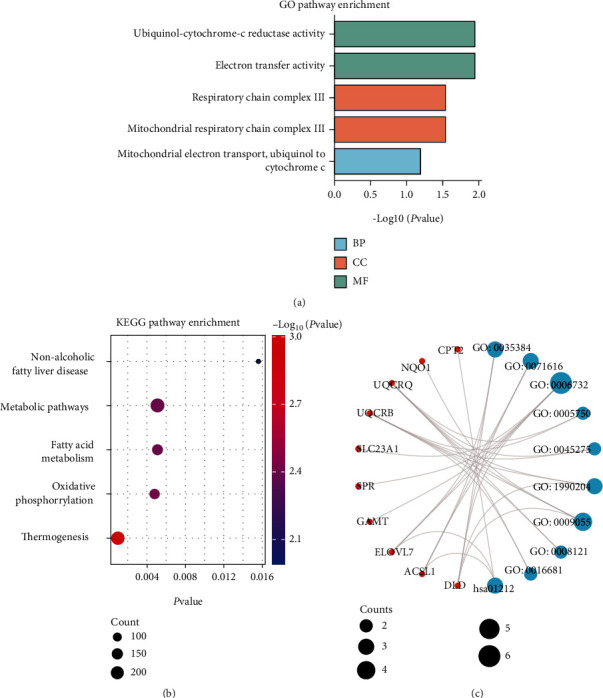
Construction of DEGs' GO and KEGG network and identification of hub genes. (a) Top 5 terms in GO pathway enrichment results of upregulated and downregulated DEGs separately. Blue charts: downregulated DEGs enrichments. Red charts: upregulated DEGs enrichments. (b) Top 5 terms in KEGG pathway enrichments results of upregulated DEGs. (c) A total of 10 hub genes via GO and KEGG network. GO: Gene Ontology; KEGG: Kyoto Encyclopedia of Genes and Genomes; BP: biological process; CC: cellular component; MF: molecular function.

GO enrichment analysis and KEGG pathway analysis was carried out to elucidate the biological functions and pathways of the upregulated DEGs. The GO analysis demonstrated that upregulated DEGs were significantly enriched in mitochondrial-related terms (Figure 3(a)), such as mitochondrial electron transport, respiratory chain complex III, and electron transfer activity. According to the GO analysis, the KEGG pathway analysis of upregulated DEGs (Figure 3(b)) also proved that they participated in many energy metabolism pathways. The protein-protein interaction (PPI) network among the DEGs also indicated that mitochondrial-related genes play hub gene roles (Figures S2(a)–S2(b)). To further investigate which part of genes plays the most critical role, the GO and KEGG network with nodes and connectivity lines was performed (Figure 3(c)). The top-ranked linker nodes were *ASCL1*, *CPT2*, *DLD*, *ELVOL7*, *GAMT*, *NQO1*, *SLC23A1*, *SPR*, *UQCRB*, and *UQCRQ*, which were defined as hub genes cluster (Figure S2C). The enrichment ID table of hub genes also demonstrated the recurring keyword “mitochondrial” (Table 2). To explore the mitochondrial-related upregulated DEGs' function, we searched the hub genes in MitoCarto 3.0 database (Supplementary Table 2). Interestingly, we found that these genes are widely related to metabolism, mitochondrial central dogma, mitochondrial dynamics and surveillance, OXPHOS, etc. During all related genes, DLD had been mostly labeled (Figure S2(d)).

### 3.3. Validate Prognostic Effect of the Hub Genes in Trastuzumab Adjuvant Therapy Cohort

**Figure 4:**
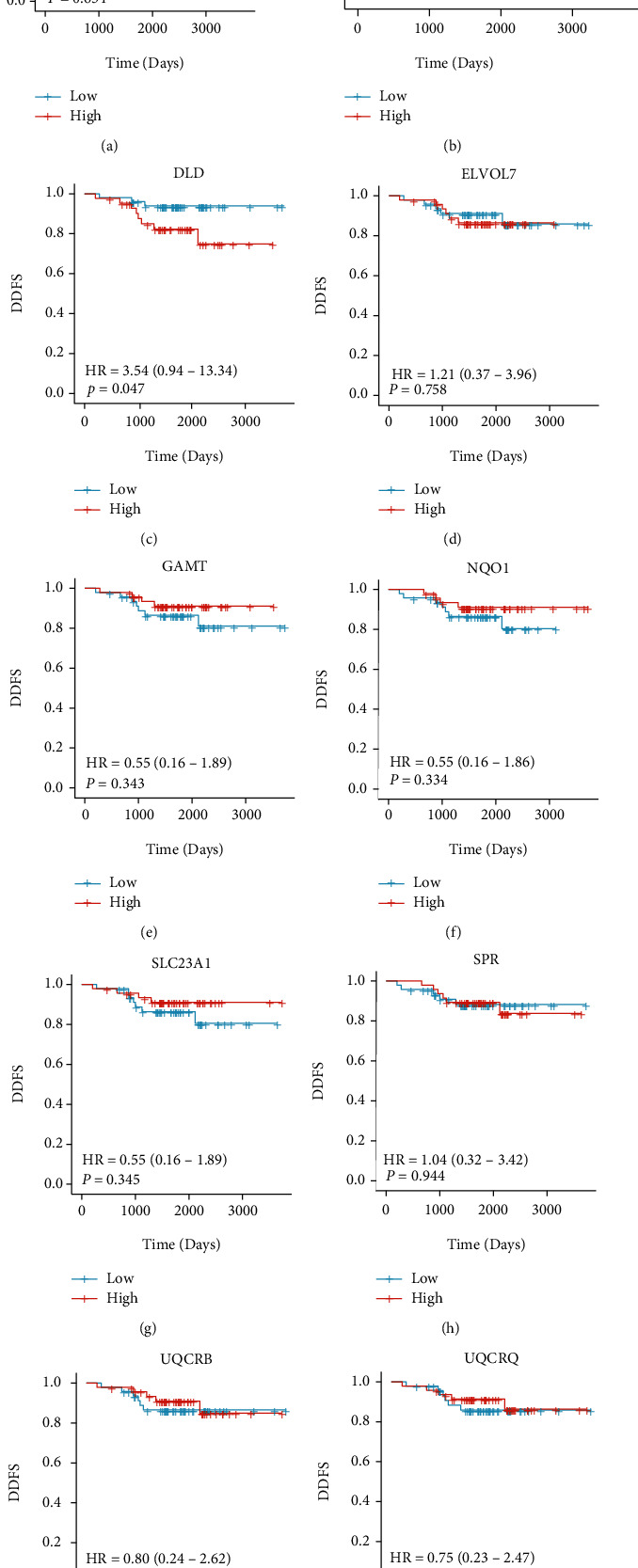
The distant disease-free survival (DDFS) analysis of 10 hub genes in GSE58984. Only patients with overexpression of DLD had a reduced DDFS compared to the low expression group (*P* < 0.05).

We further tested their prognostic robustness in an adjuvant treatment cohort to determine if the hub genes could predict the prognosis. In GSE58984, 94 patients received trastuzumab as adjuvant treatment (Supplementary Table 2). The cohort also offered DDFS information, allowing us to test hub genes' prognostic value (Figures 4(a)–4(j)). Among ten hub genes, high expression of *ACSL1*, *DLD*, *ELVOL7*, and *SPR* increased the risk of distant disease relapse. To our interest, only patients with DLD overexpression showed a significantly reduced DDFS compared to the low expression group (HR = 3.54, *P* = 0.047).

### 3.4. Explore DLD in Breast Cancer and HER2 Overexpression Subtype

**Figure 5:**
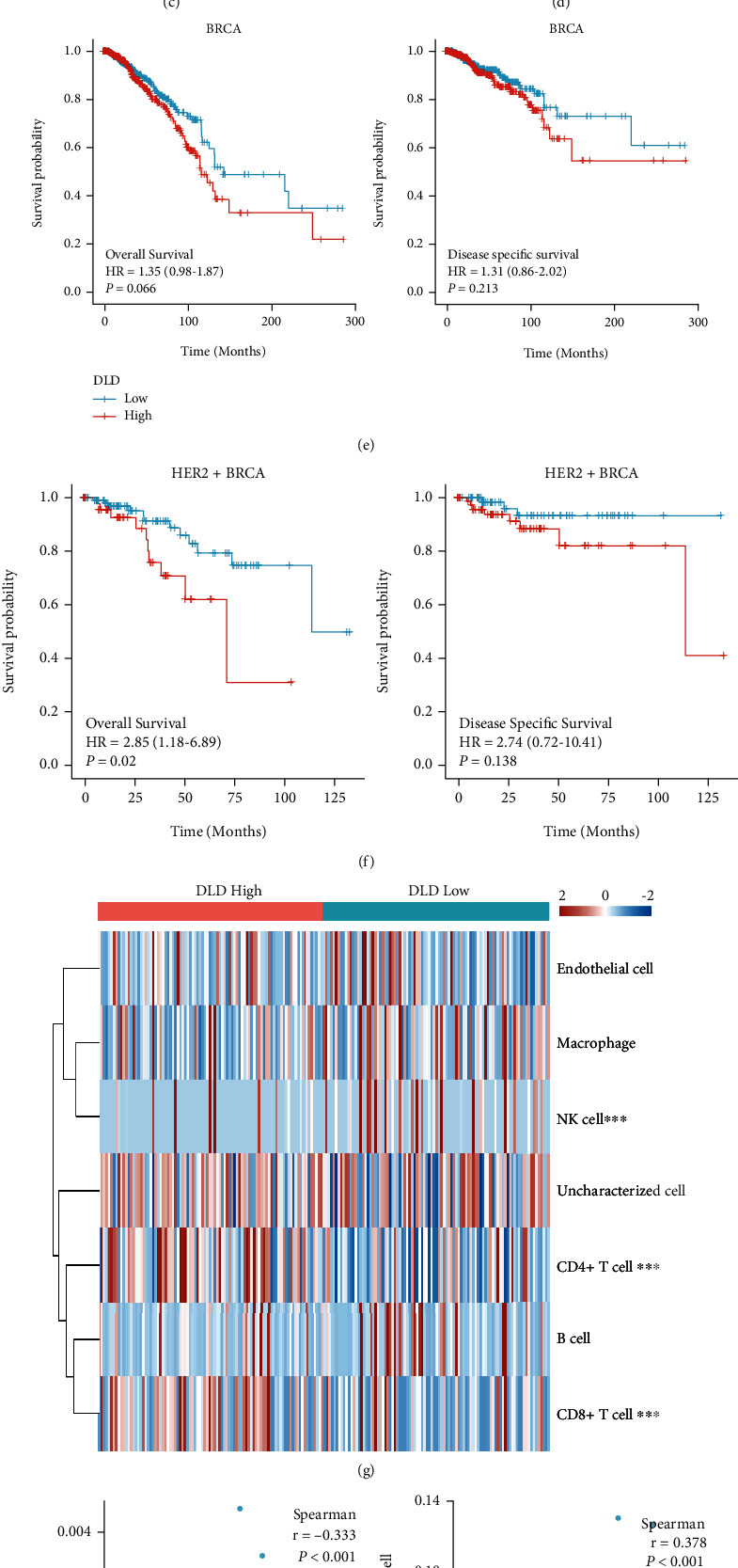
Exploration of *DLD* in clinical characteristics, survival analysis and immune analysis. (a) The expression level of *DLD* between tumor and paratumorous tissues. (b) The expression level of *DLD* among different PAM50 subtypes. (c) The expression level of *DLD* between patients ≤60 years and >60 years. (d) The expression level of *DLD* between IDC and ILC. (e) The overall survival analysis and disease-specific survival of *DLD* in BRCA of TCGA cohort. (f) The overall survival analysis, disease-specific survival, and progress-free interval of *DLD* in HER2+ BRCA of TCGA cohort. (g) The immune landscape of *DLD* in HER2+ BRCA based on EPIC immune algorithm. (h) The correlation between DLD expression and NK cells, CD4+ T cells, and CD8+ T cells. IDC: invasive ductal carcinoma; ILC: invasive lobular carcinoma; TPM: transcripts per million; ns: nonsense; ∗*P* < 0.05; ∗∗*P* < 0.01; and ∗∗∗*P* < 0.001.

To make in-depth knowledge of *DLD*, we further compared breast cancer tissues and adjacent normal tissues in TCGA breast cancer patients (Figure 5(a)). The *DLD* expression exceeded in the adjacent normal tissue than in tumorous tissues. We also made correlation analysis stratified by *DLD* expression level in the T stage, N stage, M stage, pathological stage, histological type, ER status, HER2 status, PAM50 subtypes, menopause status, and age (Table 3). As a result, *DLD* expression level was significantly associated with M stage, age, and histological type (Figures 5(b)–5(d) and S3(a)–S3(e)). In survival analysis of the TCGA cohort, the high expression of *DLD* was associated with an increased risk of death and relapse (Figures 5(e) and S3(f)). We also performed a survival analysis of *DLD*, especially in those with HER2 overexpression (Figures 5(f) and S3(g)). In particular, the survival analyses showed that *DLD* was a more sensitive index in the HER2 overexpression subtype. Moreover, we also evaluated the immune landscape of *DLD* in the EPIC algorithm. EPIC estimated scores revealed that NK cells had the most significant correlation with *DLD* expression in HER2+ BRCA (Figure 5(g)). *DLD* had a negative correlation with NK cells (*r* = −0.333, *P* < 0.001), while CD4+ T cells (*r* = 0.378, *P* < 0.001) and CD8+ T cells (*r* = 0.268, *P* < 0.001) had a positive correlation (Figure 5(h)).

## 4. Discussion

The widespread application of trastuzumab for HER2+ breast cancer patients significantly increased patients' metastatic status and prognosis. However, a considerable number of people will develop trastuzumab insensitivity or resistance. Thus, our study first discovered a 10-gene signature that could predict the pCR rate of patients who received trastuzumab as a neoadjuvant therapeutic drug. Moreover, we also found that one of 10 genes, *DLD*, could be the predictive factor for the DDFS of HER2+ patients who received trastuzumab as postoperative adjuvant therapy. Totally, our study is the first one to uncover the trastuzumab-related gene both for neoadjuvant and adjuvant trastuzumab therapy.

Our study analyzed one GEO database based on the HER2+ breast cancer tissues of patients who received single trastuzumab therapy and one GEO database based on HER2+ cell lines, which are trastuzumab-resistant. Ultimately, we identified 86 DEGs with 50 upregulated genes and 38 downregulated genes in all three GEO databases. Then, we performed GO enrichment analysis and KEGG pathway analysis using genes upregulated. The pathways shown in GO enrichment were oxidoreductase complex, mitochondrial ATP synthesis coupled electron transport, respiratory chain complex, and mitochondrial respirasome pathways. And KEGG pathway analysis involved the metabolic pathway, fatty acid pathway, and oxidative phosphorylation (OXPHOS). Tan et al. reported that glycolysis is negatively linked to trastuzumab sensitivity [8]. Yan et al. found that OXPHOS enhancement is associated with trastuzumab resistance [9]. Our work disclosed fatty acid pathway is involved in trastuzumab resistance. Other studies also reported that fatty acid metabolism-associated proteins overexpressed in HER2-positive breast cancer cell lines and tumor sample [8]. Still, the exact function and potential molecular mechanisms of fatty acid contributing to trastuzumab resistance are worth and necessary for profound investigation.

Additionally, 10 hub DEGs were further determined. *DLD* (dihydrolipoamide dehydrogenase) was negatively associated with pCR rate in HER2+ patients receiving trastuzumab neoadjuvant therapy and DDFS in HER2+ patients receiving trastuzumab as postoperative adjuvant therapy. So, *DLD* might be a trastuzumab resistance-related gene, and its function and underlying mechanism in trastuzumab resistance need further exploration. Besides, high *DLD* expression could predict the shorter overall survival in HER2+ breast cancer patients. The immune analysis also revealed that the immune microenvironment is relevant to *DLD* expression.

Based on these results, we speculate that *DLD* possibly is the key regulated gene in HER2+ breast cancer patients. *DLD*, a class-I pyridine nucleotide-disulfide oxidoreductase family member, is a mitochondrial enzyme. It is responsible for decarboxylating pyruvate to form acetyl-CoA during glucose metabolism and the production of mitochondrial adenine triphosphate (ATP) [10]. In eukaryotes, *DLD* plays an essential role in energy metabolism. *DLD* variants could cause dihydrolipoamide dehydrogenase deficiency (DLDD). People who suffer from DLDD have lactic acidosis and neurologic deterioration due to oxidative metabolism defects [11]. There only exist a few studies about the superficial effect of DLD in cancers. For example, downregulation of *DLD* in melanoma could inhibit cell proliferation by regulating energy metabolism [12], while *DLD* overexpression in head and neck cancer (HNC) cells could induce ferroptosis [13]. However, in *DLD*, as a mitochondrial-related gene, its roles in breast cancer are poorly understood. Prior studies have noted that mitochondrial dysfunction and dynamics are closely correlated with breast cancer progression and chemosensitivity [14–17]. A strong relationship between ATP synthase and resistance to HER2-targeted antibody therapies has been reported [9]. However, there is no research exploring the role of *DLD* in breast cancer, let alone HER2+ breast cancer. Therefore, exploring how *DLD* regulates mitochondrial energy metabolism to mediate trastuzumab resistance in HER2+ patients is meaningful.

To interpret our results, two limitations still need to be considered. First, our study only enrolled one GEO dataset, which includes HER2+ breast cancer patients receiving trastuzumab as neoadjuvant therapy, and one GEO dataset, which includes trastuzumab-resistant cells and normal cells. We need to enroll more GEO datasets to dig trastuzumab-resistant genes. Second, patients in the GEO dataset who received postoperative adjuvant therapy did not receive trastuzumab as a single therapeutic drug and lacked more survival-related information.

Taken together, our present work elucidates a novel gene signature that can predict the pCR rate of HER2+ breast in neoadjuvant therapy and the prognosis of HER2+ breast cancer in adjuvant treatment simultaneously. These hub genes may influence trastuzumab resistance by regulating mitochondrial-related metabolism. Moreover, our study highlighted the gene *DLD* as a diagnostic and prognostic factor and a potential target for HER2+ breast cancer treatments.

## Figures and Tables

**Table 1:** A total of 86 DEGs were picked from 2 GSE62327 and GSE10543, including 50 upregulated and 38 downregulated genes.

DEGs	Gene names
Upregulated	*ZHX2, RIDA, SPR, GAMT, LACTB2, MYRIP, TATDN1, FBXO15, CRIP1, MYBL1, MRPL15, NPRL2, ECI2, ELOVL7, TUBAL3, ZCCHC10, UPK1A, SLC23A1, MED11, RAB27B, DCAF10, GFRA1, DLD, RIMS2, TMEM150A, IK, PMAIP1, EIF1AX, UQCRB, PTPRN2, RNF125, NQO1, INPP4B, PJA2, USP38, ARHGAP5, UQCRQ, SMIM19, CPT2, SSTR1, CSTF2T, SLC25A46, ESCO1, ACSL1, TFEC, BOLA1, COLEC12, TM2D2, SLAIN1, STOX1*
Downregulated	*LTF, SLC4A11, OSMR, SLC13A3, BPIFB2, PCDHB4, RBP1, CSRP2, PLA2G5, ATP11A, RDH10, KANK1, MAGIX, HSD17B6, ZNF350, SZT2, LTBP1, PGM1, GMDS, SCMH1, ARHGAP44, SLPI, MBNL2, FIGN, SUSD1, ZNF341, TRIM38, LIMK1, PTPN14, FZD1, RASAL2, EGFR, RAPH1, COL26A1, KCTD7, GALNT12, CDCA7L, COPS7B*

DEGs: differentially expressed genes.

**Table 2:** GO and KEGG analysis terms of DEGs in network.

Ontology	ID	Description	Gene ratio	Gene
BP	GO:0035384	Thioester biosynthetic process	3/44	*DLD, ACSL1, ELOVL7*
BP	GO:0071616	Acyl-CoA biosynthetic process	3/44	*DLD, ACSL1, ELOVL7*
BP	GO:0006732	Coenzyme metabolic process	6/44	*DLD, ACSL, GAMT, SPR, SLC23A1, ELOVL7*
CC	GO:0005750	Mitochondrial respiratory chain complex III	2/45	*UQCRB, UQCRQ*
CC	GO:0045275	Respiratory chain complex III	2/45	*UQCRB, UQCRQ*
CC	GO:1990204	Oxidoreductase complex	3/45	*DLD, UQCRB, UQCRQ*
MF	GO:0009055	Electron transfer activity	4/43	*NQO1, DLD, UQCRB, UQCRQ*
MF	GO:0008121	Ubiquinol-cytochrome-c reductase activity	2/43	*UQCRB, UQCRQ*
MF	GO:0016681	Oxidoreductase activity, acting on diphenols and related substances as donors, cytochrome as acceptor	2/43	*UQCRB, UQCRQ*
KEGG	hsa01212	Fatty acid metabolism	3/23	*CPT2, ACSL1, ELOVL7*

**Table 3:** Correlation between the expression level of *DLD* and clinicopathological characteristics in BRCA.

Characteristic	Low expression of DLD	High expression of DLD	*P*
*n*	541	542	
T stage, *n* (%)			0.060
T1	135 (12.5%)	142 (13.1%)	
T2	311 (28.8%)	318 (29.4%)	
T3	83 (7.7%)	56 (5.2%)	
T4	12 (1.1%)	23 (2.1%)	
N stage, *n* (%)			0.541
N0	262 (24.6%)	252 (23.7%)	
N1	168 (15.8%)	190 (17.9%)	
N2	60 (5.6%)	56 (5.3%)	
N3	41 (3.9%)	35 (3.3%)	
M stage, *n* (%)			0.019
M0	436 (47.3%)	466 (50.5%)	
M1	8 (0.9%)	12 (1.3%)	
Pathologic stage, *n* (%)			0.540
Stage I	95 (9%)	86 (8.1%)	
Stage II	301 (28.4%)	318 (30%)	
Stage III	129 (12.2%)	113 (10.7%)	
Stage IV	8 (0.8%)	10 (0.9%)	
Histological type, *n* (%)			< 0.001
Infiltrating ductal carcinoma	355 (36.3%)	417 (42.7%)	
Infiltrating lobular carcinoma	130 (13.3%)	75 (7.7%)	
ER status, *n* (%)			0.204
Negative	110 (10.6%)	130 (12.6%)	
Indeterminate	1 (0.1%)	1 (0.1%)	
Positive	410 (39.6%)	383 (37%)	
HER2 status, *n* (%)			0.405
Negative	272 (37.4%)	286 (39.3%)	
Indeterminate	8 (1.1%)	4 (0.6%)	
Positive	81 (11.1%)	76 (10.5%)	
PAM50, *n* (%)			0.105
Normal	27 (2.5%)	13 (1.2%)	
LumA	301 (27.8%)	261 (24.1%)	
LumB	91 (8.4%)	113 (10.4%)	
HER2	40 (3.7%)	42 (3.9%)	
Basal	82 (7.6%)	113 (10.4%)	
Menopause status, *n* (%)			0.154
Pre	103 (10.6%)	126 (13%)	
Peri	19 (2%)	21 (2.2%)	
Post	367 (37.8%)	336 (34.6%)	
Age, median (IQR)	60 (50, 69)	56 (48, 65)	0.002

## Data Availability

The data used to support the findings of this study are included within the article and supplementary files.

## Supplementary Materials

Supplementary Materials Figure S1. Forest plots to demonstrate the results of the univariate Cox regression analysis between DEGs expression and OS. Figure S2. PPI network of DEGs. Figure S3. Correlation between clinicopathological characteristics and the expression of *DLD* and survival analysis of *DLD* in TCGA. Supplementary Table 1. Clinical characteristics of the patients in GSE62327. Supplementary Table 2. MitoPathway analysis of mito-related genes in upregulated DEGs. Supplementary Table 3. Clinical characteristics of the patients in GSE58984.
