# Supplementary material for: Identification of Genes Predicting Poor Response of Trastuzumab in Human Epidermal Growth Factor Receptor 2 Positive Breast Cancer
Source: J Immunol Res. 2022 Jul 27;2022:9529114. doi: 10.1155/2022/9529114 (PMC9348965; doi:10.1155/2022/9529114)
Supplement: Supplementary Materials — Figure S1. Forest plots to demonstrate the results of the univariate Cox regression analysis between DEGs expression and OS. Figure S2. PPI network of DEGs. Figure S3. Correlation between clinicopathological characteristics and the expression of DLD and survival analysis of DLD in TCGA. Supplementary Table 1. Clinical characteristics of the patients in GSE62327. Supplementary Table 2. MitoPathway analysis of mito-related genes in upregulated DEGs. Supplementary Table 3. Clinical characteristics of the patients in GSE58984. [file 9529114.f1.zip › Supplementary figure legends (1).docx]

**Supplementary figure legends**

**Figure S1 Forest plots to demonstrate the results of the univariate Cox regression analysis between DEGs expression and OS**

A. Forest plot of 50 up-regulated DEGs.

B. Forest plot 38 down-regulated DEGs.

**Figure S2 PPI network of DEGs**

A. The online STRING database constructed the PPI network between 50 up-regulated DEGs.

B. The online STRING database constructed the PPI network between 38 down-regulated DEGs.

C. The hub genes PPI network conducted by cytoscape.

D. Word clouds of mitochondrial-related genes.

PPI, protein-protein interaction.

**Figure S3 Correlation between clinicopathological characteristics and the expression of *DLD* and survival analysis of *DLD* in TCGA**

A. The expression level of *DLD* between HER2+ tissues and HER2- tissues.

B. The expression level of *DLD* among different T stages.

C. The expression level of *DLD* among different N stages.

D. The expression level of *DLD* between different M stages.

E. The expression level of *DLD* among different pathological stages.

F. The progress-free interval of *DLD* in BRCA of TCGA cohort.

G. The progress-free interval of *DLD* in HER2+ BRCA of TCGA cohort.
