# Supplementary material for: Identification of Genes Predicting Poor Response of Trastuzumab in Human Epidermal Growth Factor Receptor 2 Positive Breast Cancer
Source: J Immunol Res. 2022 Jul 27;2022:9529114. doi: 10.1155/2022/9529114 (PMC9348965; doi:10.1155/2022/9529114)
Supplement: Supplementary Materials — Figure S1. Forest plots to demonstrate the results of the univariate Cox regression analysis between DEGs expression and OS. Figure S2. PPI network of DEGs. Figure S3. Correlation between clinicopathological characteristics and the expression of DLD and survival analysis of DLD in TCGA. Supplementary Table 1. Clinical characteristics of the patients in GSE62327. Supplementary Table 2. MitoPathway analysis of mito-related genes in upregulated DEGs. Supplementary Table 3. Clinical characteristics of the patients in GSE58984. [file 9529114.f1.zip › Supplementary Table 1 (1).docx]

**Supplementary Table 1 Clinical characteristics of the patients in GSE62327**

| Characteristic | levels | Numbers |
| --- | --- | --- |
| n |  | 24 |
| Tumor grade, n (%) | T1 | 2 (8.3%) |
|  | T2 | 14 (58.3%) |
|  | T3 | 7 (29.2%) |
|  | T4 | 1 (4.2%) |
| Lymph nodes status, n (%) | Negative | 12 (60%) |
|  | Positive | 8 (40%) |
| Histological grade, n (%) | I | 1 (4.2%) |
|  | II | 4 (16.7%) |
|  | III | 19 (79.2%) |
| Response, n (%) | CR | 6 (25%) |
|  | PR | 18 (75%) |
